# Complementary techniques for the reliable characterisation of tissue samples: A case study on pancreatic tumours analysed by means of X-ray fluorescence analysis and IR spectroscopy

**DOI:** 10.1371/journal.pone.0306795

**Published:** 2024-09-04

**Authors:** Katja Frenzel, Yves Kayser, Andrea Hornemann, Bernd Kästner, Arne Hoehl, Petros Mouratidis, Ian Rivens, Gail ter Haar, Burkhard Beckhoff

**Affiliations:** 1 Physikalisch-Technische Bundesanstalt, Berlin, Germany; 2 Therapeutic Ultrasound, Division of Radiotherapy and Imaging, The Institute of Cancer Research, London, United Kingdom; K Ramakrishnan College of Technology, INDIA

## Abstract

An improvement in the reliability and comparability of tissue characterization results is crucial for enabling further progress in cancer detection and the assessment of therapeutic effects. This can only be achieved by integrating quantitative methods into well-established qualitative characterization routines. This case study presents a hybrid metrological approach for tissue characterisation including vibrational Fourier Transform InfraRed (FTIR) spectroscopy and traceable reference-free X-Ray Fluorescence analysis (XRF). Through the combination of spatially resolved qualitative molecular information with quantitative elemental concentrations an all-encompassing sample characterisation can be provided. The study was performed on tissue sections of syngeneic murine pancreatic ductal adenocarcinoma KPC (Kras^G12D/+^; Trp53^R172H/+^; Pdx-1-Cre) tumours *ex-vivo*. Sections from healthy pancreatic tissues, sham-exposed tumours and tumours subjected to low dose radiotherapy treatment (2 Gray and 6 Gray) were analysed using both methods. Additional sample integrity studies using Near Edge X-ray Absorption Fine Structure (NEXAFS) spectroscopy at the carbon and nitrogen K-edges were performed to assess the effect of sample aging and XRF investigations on the samples. Results showed an increase in the concentrations of elemental biomarkers, including S, K and amide I structures in malignant pancreatic tissue compared to healthy pancreatic tissue. The exposure of tumours to 6 Gy radiation decreases the levels of these elements towards a phenotype seen in the healthy pancreas. A protocol for hybrid investigations is presented, with emphasis on the sample preparation, minimizing the impact of consecutive applied methods on their measurands, and ensuring the compatibility and reliability of achieved results. The study demonstrates the cancer recognition capabilities, and the sensitivity for low dosage radiotherapy treatment monitoring for each method individually and assesses the potential of combining molecular fingerprinting with non-destructive quantitative elemental information for tissue sample characterization.

## Introduction

Cancer is responsible for more than 1.9 million deaths per year in the European Union [1]. The 5-year survival rates for patients with pancreatic cancer are among the worst of any cancer type and have effectively remained unchanged over the past 20 years. One reason for this dismal prognosis is the lack of methods for detecting pancreatic tumours at early stages. The reliable detection of cancer, and the assessment of therapeutic success through the monitoring of quantifiable parameters is fundamental to ensuring the best and most efficient treatment for patients. Currently, most cancers are diagnosed by pathologists using microscopic evaluation of stained tissue samples. This approach is only successful, if cancerous or precancerous lesions can be identified, which means that significant genetic changes must already be present in the tissue. The method involves the complex and time-consuming process of histochemical staining of tissue samples, and its sensitivity depends heavily on the experience of the pathologist [2, 3] as well as on the sample preparation itself [4]. If tumours are histologically similar to the tissue from which they originated, or the cancer cells are poorly differentiated, this approach leads to inconclusive results [5]. Additional methods are based on the detection of a variety of cancer-type-related molecular biomarkers, and growth factors in tissues, cells, and body fluids. Biomarkers, defined as disease-related molecular changes in body fluids and tissues [6], are essential in facilitating screening and diagnosis. Although the research and application of cancer-type and cancer-stage specific molecular biomarkers are progressing rapidly [7], the overwhelming number of potential cancer-indicating molecules is making reliable detection difficult [8], leading to a high number of false positive or false negative diagnostic results. Another major challenge hindering the clinical applicability of complex molecular biomarkers lies in the lack of both the comparability and the reproducibility of results due to the variation between sample preparation assays, employed materials and data acquisition and processing procedures [9]. To ensure the comparability across laboratories and batches relative quantitative techniques should be complemented by absolute quantitative methods that can provide a reference value with related uncertainty [10]. Thus, the incorporation of elemental biomarkers into cancer diagnostics and therapy could provide additional molecule-independent information, which could lead to a more accurate assessment of the tissue sample, and ultimately to better patient treatment.

In order to achieve a complete molecular and elemental characterisation of a sample, multi-method investigations must be performed. Fourier transform infrared (FTIR) spectroscopy is a standard tool for the characterisation of molecular structures. It is a surface sensitive and non-destructive technique with a spatial resolution in the μm range, which may be further enhanced by super-resolution techniques [11]. The FTIR technique is being used increasingly as a potential diagnostic tool [12–16]. By employing mid-infrared (MIR) spectral data, it is possible not only to assess the chemical structures but also to achieve differentiation based on vibrational spectroscopic signatures supported by multivariate statistical analysis [17]. With the development of focal-plane-array (FPA) detector-based imaging modalities, new applications have emerged, with the focus on cancer research, biology and chemical engineering, in order to unravel the structural and chemical-physical propensities at molecular level [18].

The potential of FTIR spectroscopy as a cancer diagnostic tool has been recognised for decades, allowing as it does, identification of suitable biomarkers for the purpose of cancer identification [19], monitoring chemotherapy [20] and radiation exposure [21, 22] and other therapy effects by following cellular repair processes [23]. Examples are the detection of alterations in protein conformation and composition [24] in ovarian cancer cell lines or radiation-induced apoptosis in human lymphocytes [25]. Recently, and more closely related to this study, human pancreatic biopsies have been investigated using FTIR hyperspectral imaging [26], and spectral markers of certain lesions have been suggested. A recent review of FTIR spectroscopy as a diagnostic method for several different types of cancer has been published [20]. This, also highlights the improvement in sensitivity and specificity over conventional clinical analysis in several cases.

X-ray fluorescence analysis (XRF) is a non-invasive method and is already widely used for contamination monitoring and quantification of trace elements in environmental science [27, 28], microelectronics [29–32], archaeometry [33, 34] and geology. The method uses characteristic XRF radiation emitted from a sample when excited typically with X-ray radiation. The characteristic XRF energies are element- and transition-specific, as electrons from different atomic shells can be involved in the process [35]. Using the X-ray intensities detected at the characteristic XRF line energies, the elemental composition and speciation of a sample can be determined. Using (radiometrically) calibrated instrumentation and a good knowledge of atomic fundamental parameters, XRF can provide quantitative (in a physical traceability chain) elemental information in the reference-free approach. This method does not need any reference materials or calibration samples [36, 37], as it is common in a chemical traceability chain. Reference-free XRF (RF-XRF) enables the quantification of a wide range of elements, while minimal, to no, prior information about the composition of the samples is required [29, 34, 38–40]. Furthermore, XRF has minimal requirements for sample preparation. The sample does not need to be stained, labelled, or embedded.

The main objective of this study was to investigate these two analytical methods in terms of their discrimination capability and quantification reliability for biological tissues. The potential and the drawbacks of an all-encompassing analysis of identical samples by RF-XRF and FTIR were assessed. These techniques were applied to a small ensemble of murine pancreatic ductal adenocarcinoma (PDAC) KPC (Kras^G12D/+^; Trp53^R172H/+^; Pdx-1-Cre) tumours. In addition to the usage of SI (international system of units) traceable XRF and IR spectroscopy, this case study involved the assessment of multimodal/hybrid metrology approaches to biological samples. It has investigated synergies resulting from combining elemental and molecular biomarkers to discriminate between healthy and cancer tissue, and monitoring the response of cancer to radiotherapy.

## Materials and methods

### Cell lines and in vivo model

KPC cells were maintained as a sub-confluent monolayer at 37°C in 175 cm^2^ flasks in a humidified atmosphere containing 5% CO_2_. They were passaged using Dulbecco’s Modified Eagles medium (DMEM) supplemented with 2mM L-glutomanine and 10% Foetal bovine serum (FBS). Screening for mycoplasma contamination was carried out before in vivo injection of cells, and cells were STR profiled. Syngeneic PDAC tumours were grown after subcutaneous injection of 2 ⋅ 10^6^ KPC cells (Kras^G12D/+^; Trp53^R172H/+^; Pdx-1-Cre) into the flank of immune competent C57BL/6 mice. All studies were performed with approval from the UK Home Office, and the Institute of Cancer Research (ICR) local Animal Welfare and Ethics Committee. At no instance, the experiments exceeded the severity limits allowed. The smallest possible species was used. Animal grew in our facilities receiving care 24 hours a day every day. To minimise the burden on animals, the cells were directly injected subcutaneously, thus minimising pain and the time the animals were kept in captivity. In our experience this procedure has only mild impact on the animals and full recovery is rapid. The tumours grew to the smallest volume we could successfully target (<5mm). All animals were monitored closely to ensure provision of effective pain relief (vetergesic/medetomidine). Weight, animal posture and behaviour were measured/scored daily under the FELASA guidelines, and no animal showed any signs of deterioration of their quality of life. Main methods of anaesthesia were the inhalation of isoflurane or IP injection of fentanyl/hypnovel/medetomidine. Animals were sacrificed using humane methods.

Subjects were randomized between treated and control groups, in 2 cages and subjects from either cage were exposed with 2 Gy and 6 Gy. Radiotherapy was delivered in subjects, anaesthetized by inhalation anaesthesia (isoflurane), using a dedicated small animal image guided radiotherapy system (SARRP, Xstrahl Ltd, UK) using two opposing beams, in a single fraction. The dose planning, based on the cone-beam CT scan of the animal (0.25 mm resolution, acquired at 60 keV, 0.8 mA, with 1 mm aluminum filtration as part of the SARPP’s on-board imaging facility, approximate imaging dose was 0.1 Gy) was performed using the MuriPlan software (SARRP, Xstrahl ltd., UK) [41]. All treatments were planned individually. Air, lung, fat, muscle and bone were segmented for treatment planning. No individual contouring was attempted. Dose iso-centres were placed close to the centre of the tumour but offset to avoid unnecessary exposure of abdominal tissue. Superposition convolution was used to check that the planned dose was delivered to the whole tumour. Treatment was delivered using a 10x10 collimator to minimize dose outside the target tumour and using 2 parallel opposed beams at an angle optimized for tumour coverage. Radiation was delivered as anticipated, and a record of irradiations was taken and can be seen in S1 Table. After irradiation, subjects were recovered on a heating mat until fully conscious before being returned to the housing facility. Sham treated animals were also anaesthetised for approximately the same time as the radiation treated cohort, but were not CT imaged or irradiated. ICR was responsible for the allocation and conduct of the treatments, whereas Physikalisch-Technische Bundesanstalt (PTB) was responsible for the processing of the tissue samples, assessment, and data analysis.

Radiotherapy in a single fraction was delivered to these tumours using a dedicated small animal image guided radiotherapy system (SARRP, Xstrahl Ltd, UK) using two opposing beams. The dose planning, based on a cone-beam CT scan of the animal, was performed using the *MuriPlan^©^* software. KPC tumours were exposed to 2 Gy or 6 Gy, or they were sham treated. They were excised 3 or 12 days after irradiation. The healthy pancreases of animals were also excised. Samples were snap frozen immediately after excision by immersion in liquid nitrogen, and kept on dry ice until 10 *μ* m sections were cut using a cryotome. At least 2 consecutive sections were placed on *MirrIR Corner Frosted* glass slides from *Kevley Technologies^©^*. Thereafter the tissues were left to air-dry. An overview of the sample treatments is given in Table 1.

**Table 1:** Overview of cryo-sectioned samples investigated by RF-XRF and FTIR. Murine subcutaneous PDAC KPC tumours were investigated. Prior to drying, the sections of tumours were placed on coated glass slides from *Kevley Technologies^©^*, to allow FTIR investigation.

Tissue type	Abbreviation	Treatment dose	Time of excision after treatment
		(Gray)	(Days)
Healthy pancreas	HP	0	N/A
Pancreatic tumour	PC	0	N/A
PDAC KPC tumour	2Gy-D3	6 Gy	12 d
PDAC KPC tumour	2Gy-D12	6 Gy	3 d
PDAC KPC tumour	6Gy-D3	2 Gy	12 d
PDAC KPC tumour	6Gy-D12	2 Gy	3 d

### Fourier-transform infrared spectroscopy

Absorbance spectra of cryo-sectioned pancreas tissues were recorded in reflection geometry in the MIR spectral range of 3900 cm^-1^ and 900 cm^-1^. The experiments were performed using a Vertex 80v FTIR spectrometer (Bruker Optics GmbH) to which a FTIR Hyperion 3000 microscope was coupled. The spectrometer was fitted with a KBr beamsplitter and a globar was implemented as a radiation source for microspectroscopic investigations. For data acquisition, a LN_2_-cooled multi-element mercury cadmium telluride detector, a FPA detector, with 128^2^ pixel elements and a spectral resolution of 4 cm^–1^ was used. Micro-spectroscopic experiments on thin microtome tissue sections were conducted with a Cassegrain objective at a 15× magnification, enabling the study of a sample area of 345 *μ*m^2^ with approximately 2.87 *μ*m lateral resolution; the latter corresponds to the dimension of a single pixel.

Each spectrum was collected with the Opus software v.7.2 (Bruker Optics GmbH) and consisted of 128 averaged scans for pancreatic tissue samples. All interferogram scans were submitted to a Blackman Harris 3-term window function and to a zerofilling factor of 2 prior to Fourier transformation. Background scans were collected prior to each sample measurement from a region free of samples, here on a clean low-e-slide, and compared with the sample spectrum. From the FTIR micro-spectroscopic datasets comprising 16385 spectra, respectively, 900 spectra were extracted and their arithmetic means and standard deviations calculated.

### Reference-free X-ray spectrometry

All XRF measurements were performed after FTIR analysis of the samples. RF-XRF measurements for the quantification of P, S, Cl, and K in the different tissue sections were acquired at the four-crystal-monochromator (FCM) beam line [42]. Sample stability investigations by Near Edge X-ray Absorption Fine Structure (NEXAFS) spectroscopy for light elements (C, N) were performed at the plane grating monochromator (PGM) beam line [43, 44]. Both synchrotron-radiation beam lines are located in the PTB laboratory at the BESSY II electron storage ring. Additional investigations of the KPC samples were performed to verify the quantifiability of Na and Mg (at the PGM beam line, incident photon energy of 1.7 keV) as well as from Ca to Zn (at the FCM beam line, incident photon energy of 10.4 keV). The high levels of partially inhomogeneously distributed Zn, Mg, Ca, Mn, Cu in addition to other elements inside the substrate prevented reliable quantification of these elements.

**Fig 1:**
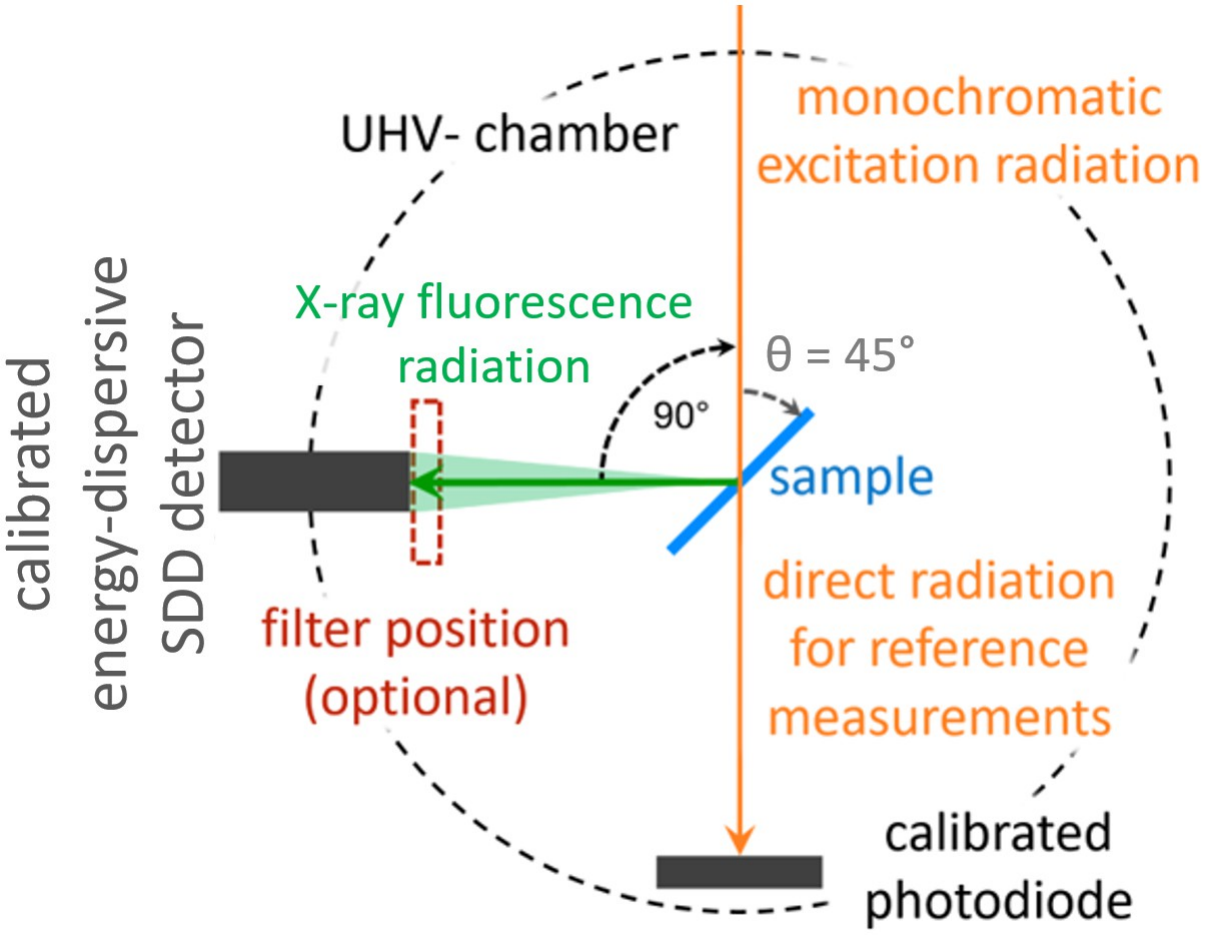
Schematic layout of the positioning of calibrated instrumentation relative to the excitation radiation and the samples inside the UHV-chamber for RF-XRF and NEXAFS experiments.

For all these measurements the end station was an ultrahigh-vacuum chamber equipped with a 9-axis manipulator [45] (see Fig 1). Within this end station the samples can be positioned precisely and oriented with respect to the incident synchrotron radiation. The X-ray beam spot size on the sample was around 300 *μ*m horizontally and 150 *μ*m vertically at the PGM beam line and around 300 *μ*m horizontally and 180 *μ*m vertically at the FCM beam line. The beam dimensions determined the step size for the mapping scans, in which the spatial distribution of the different elements in each sample was assessed. For the selection of the horizontal step size, the samples under investigation were taken to be oriented at 45° with respect to incident synchrotron radiation. An energy-dispersive Silicon Drift Detector (SDD) was positioned perpendicular to the propagation direction and within the polarization plane of the linearly polarized incident X-ray beam to minimize scattered radiation [46]. The SDD used was calibrated in terms of response behaviour [47] and detection efficiency [48].

#### Element quantification

The fluorescence spectra were deconvolved using the detector response functions for the detected fluorescence lines and background contributions [49] in order to access the resulting event rate *N*_*i*_ for each fluorescence line. To derive the absolute emitted fluorescence intensity, *N*_*i*_ is normalized with respect to the sine of the X-ray incident angle *θ*, the incident photon flux Φ_0_, the energy-dependent detection efficiency *ϵ*(*E*) of the SDD for the respective fluorescence photons [48] and the effective solid angle [50] of detection with respect to $\frac{\Omega }{4\pi }$. Φ_0_ was measured using radiometrically calibrated photodiodes. The determination of Ω strongly depends on accurate knowledge of the detection geometry as well as of the incident beam profile [51, 52]. From the absolute XRF intensity the elemental concentration *C*_*i*_, defined as mass per unit volume, can be extracted for each measured position of the sample using the Sherman equation [53]:

$$\begin{array}{c} C_{i}=\frac{4\pi \;N_{i}\left(E_{i}\right)\;d}{\Phi_{0}\left(E_{0}\right)\;\epsilon \left(E_{i}\right)\;\Omega \;\gamma_{i,X}\;g_{i,X,Y}\;\tau_{i,X}\left(E_{0}\right)}\;\frac{(\mu_{s}\left(E_{0}\right)+\mu_{s}\left(E_{i}\right))\;\rho d}{1-\text{exp}\;(-\frac{\mu_{s}\left(E_{0}\right)+\mu_{s}\left(E_{i}\right)}{\text{sin}(\theta )}\rho d)} \end{array}$$ (1)

An overview of the variables and their units is provided in Table 2. The first part of the equation combines instrumental parameters and the element dependent physical fundamental parameters [54]. The second part of Eq 1 is an absorption correction factor which includes the angle of the incident beam *θ*_in_ and the detection angle *θ*_out_ with respect to the surface of the sample, and hence addresses the absorption of the incident beam during excitation as well as the absorption of the fluorescence on its way to the surface of the sample. The absorption correction factor includes sample density *ρ* [55] and thickness *d*, as well as the mass attenuation coefficient *μ*_*s*_(*E*).

**Table 2:** Overview of the variables (and their corresponding units) used in Eq 1 for the reference-free elemental quantification by XRF.

Variable	Meaning	Unit
*C*_*i*_	elemental concentration	g cm^-3^
Φ_0_	incident photon flux	s^-1^
Φ_*i*_	emitted x-ray fluorescence	s^-1^
*N*_*i*_	x-ray fluorescence count rate	s^-1^
*ϵ*(*E*)	detector efficiency [48]	
Ω	solid angle of detection [50]	sr
*γ*_*i*_	fluorescence yield [54]	
*g*_*i*,*X*,*Y*_	transition probability [54]	
*τ*_*i*,*X*_(*E*)	photoionization cross section [54]	cm^2^ g^-1^
*μ*(*E*)	mass attenuation coefficient [54]	cm^2^ g^-1^
*ρ*	sample density [55]	g cm^-3^
*d*	sample thickness	cm

#### Sample roughness correction

**Fig 2:**
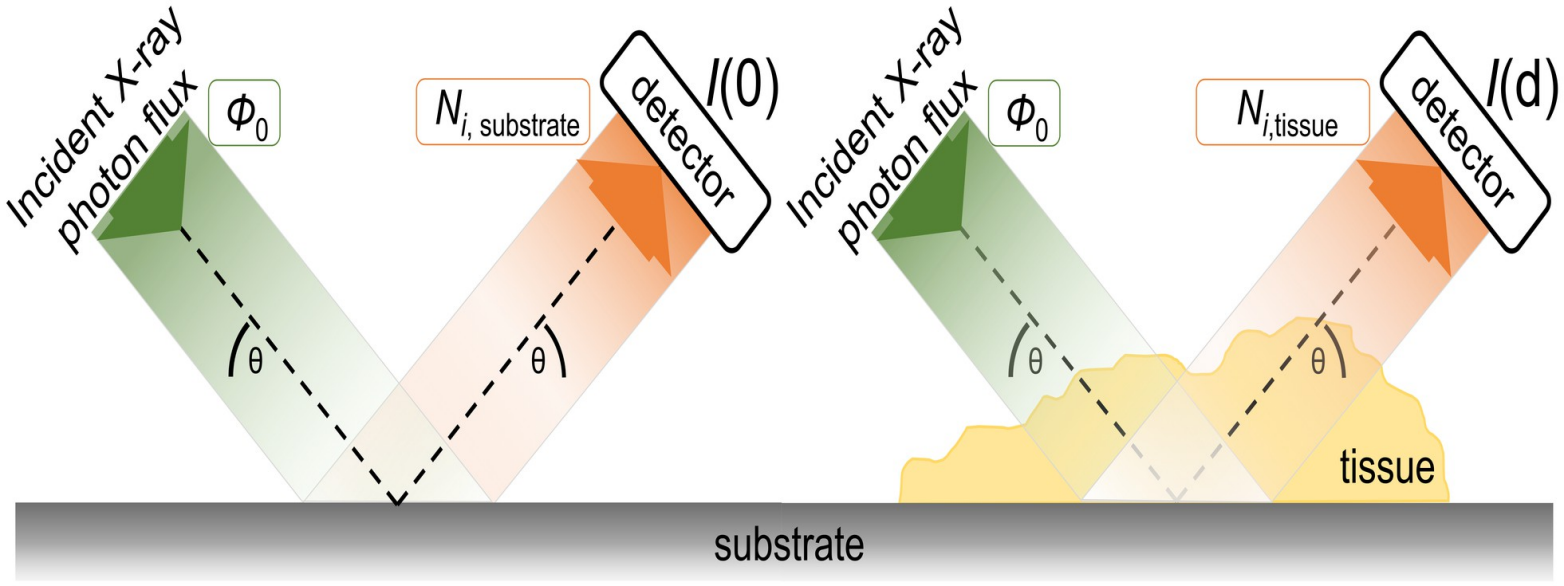
Schematics of the excitation beam (green) and detected fluorescence radiation (orange) paths during measurements of the substrate (left) and tissue (right) used for spatially resolved sample thickness determination.

RF-XRF provides mass per unit area information about the elemental mass deposition of an element inside the sample of interest, which can be understood as the integration (in-depth) of the number of atoms contained locally in the tissue. Dried tissue sections have local thickness variations. Hence, the concentration of an element, described as a thickness-independent quantity, allows better comparability between the tissues probed, and addresses effects of intra-tissue roughness. Additionally, the determination of the sample thickness is crucial for accurate quantification of (especially light) elements (see Eq 1), due to the varying information depth of element specific X-ray fluorescence. Therefore, the local sample thickness had to be obtained for each measured position in the sample. Standard transmission measurements for thickness determination could not be performed since the tissue sections could not be prepared as free-standing samples and the supporting substrates used were too thick due to the substrate requirements for FTIR. To this end, a routine to derive thickness information simultaneously with the quantification of elemental concentrations had to be developed. The Si K*α* fluorescence intensity originating in the substrate below the sample was measured simultaneously with the elements inside the sample (see Fig 2). The incident photons and the Si K*α* fluorescence photons had to cross the sample *N*_*i*,*tissue*_. Therefore, the characteristic Si XRF radiation could be used to calculate the thickness of the sample by applying Lambert-Beers law using the assumption *θ*_in_ = *θ*_out_ = *θ* = 45° (see Eq 2 below). Additionally, measurements of the substrate were performed in order to determine the fluorescence intensity *N*_*i*,*substrate*_ without sample related absorption.

$$\begin{array}{c} I\left(d\right)=I_{0}\;\text{exp}\;(-\frac{\mu_{s}\left(E_{0}\right)+\mu_{s}\left(E_{i}\right)}{\text{sin}(\theta )}\rho d)\;\Rightarrow \;d=-\text{ln}\;(\frac{I(d)}{I_{0}})\frac{\text{sin}(\theta )}{(\mu_{s}\left(E_{0}\right)+\mu_{s}\left(E_{i}\right))\rho }, \end{array}$$ (2)

where $I_{0}=\frac{\Phi_{i,\text{substrate}}}{\Phi_{0,\text{substrate}}}$, $I\left(d\right)=\frac{\Phi_{i,\text{tissue}}}{\Phi_{0,\text{tissue}}}$ with $\Phi_{i}=\frac{N_{i}}{\epsilon (E)}$.

#### Statistical analysis

The elemental concentration of P, S, Cl, and K for each lateral pixel of a RF-XRF map is determined from independent measurements with an combined standard uncertainty [56]. The concentration of elements within the same tissue can vary laterally. Thus, the quantification results are shown as the median of quantified elemental concentrations of a sample ±the combined standard uncertainty of the median. Concentration distributions of tissue samples were compared using the two-sided Mann-Whitney-Wilcoxon test from *Statannotations* [57], which integrates the implementation from *scipy.stats* [58–60]. The null hypothesis was that there is no difference between the concentration distributions of P, S, Cl or K of untreated cancerous tissues (PC:n = 599) and cancerous tissues treated with radiotherapy (6Gy-D12:n = 951, 6Gy-D3:n = 306, 2Gy-D12:n = 659, 2Gy-D3:n = 557) or healthy tissue (HP:n = 271). Statistical significance of the test is denoted with asteriks as described here: ns: *p* ≤ 1, *:1 ⋅ 10^−2^ < *p* ≤ 5 ⋅ 10^−2^, **: 1 ⋅ 10^−3^ < *p* ≤ 1 ⋅ 10^−2^, ***: 1 ⋅ 10^−4^ < *p* ≤ 1 ⋅ 10^−3^, ****: *p* ≤ 1 ⋅ 10^−4^.

## Results

### FTIR spectral signatures

The spectral modes that can be detected from tissues are listed in Table 3. The 1900–1500 cm^-1^ spectral region delivers characteristic frequencies of the amide I and amide II bands which refer to molecular constituents of proteins. The amide window is followed by the so-called ‘mixed region’ (1500—1300 cm^-1^), including fatty acid bending vibrations, C-N stretching and N–H bending modes of proteins, and P = O or phenoxide stretching modes of phosphate-carrying species.

**Table 3:** Overview of relevant vibrational modes detected in the MIR spectral regime.

Observed modes/cm^-1^	Molecular vibration	Band assignments
1753–1735	*ν*(C = O), *ν*(COOH)	saturated esters
1725–1705	*ν*(C = O)	ketones,–COOH
1677–1640	*ν*(C = O) or *ν*(C = C), *ν*(C = N)	amide I
1640–1614	*δ*(N–H)	primary amide
1562–1540	*δ*(N–H), *ν*(C–N), *ν*(C = N)	NHR, secondary amine, protein, nucleic acid
1454–1400	*ν*(C–N)	primary amide
1500–1200	mixed region	proteins, fatty acids, phosphate compounds
1200–1900	polysaccharide region	phosphodiester bonds (from DNA) polysaccharides

**Fig 3:**
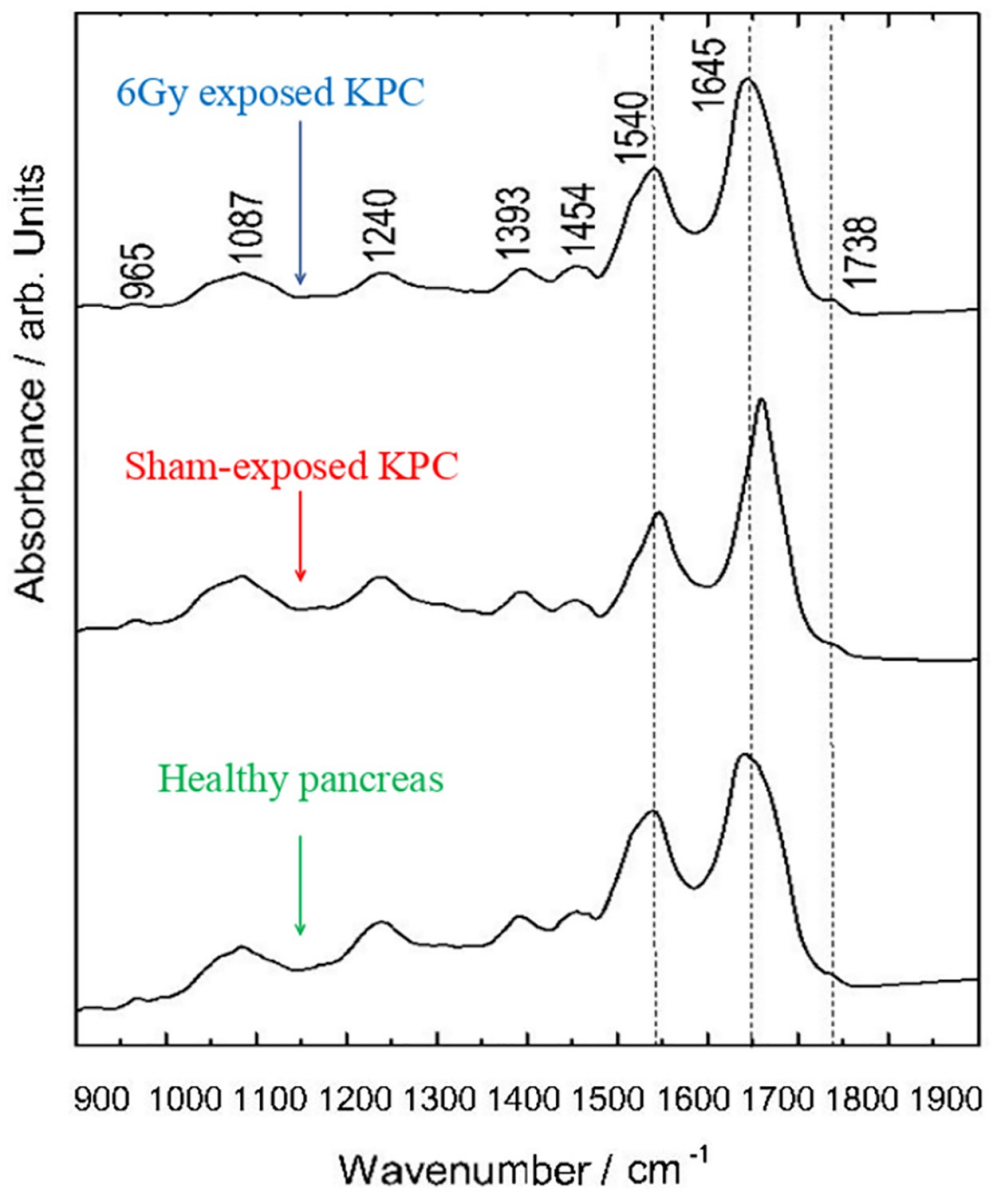
Mid-infrared spectral signatures (arithmetic means of 900 spectra) of the pancreatic samples. The dashed lines display the amide band regions with the most significant spectral changes with respect to band shifts and altered line shapes.

The IR spectral signatures of three pancreatic samples are shown in Fig 3 where healthy pancreas (HP), a sham exposed pancreatic KPC tumour (PC), as well as a KPC tumour irradiated with 6 Gy and fixed 12 days after exposure are compared. Of particular interest is the amide I band at 1645 cm^-1^, as the expected changes in protein configuration can induce meaningful variations in its shape [18, 61, 62]. The amide I band has previously been described as a convolution of the contributions arising from the various secondary structures of proteins [63]; the bands between 1620–1640 cm^-1^ and at 1690 cm^-1^ can be related to beta-sheet structures. The 1620 and 1690 cm^-1^ bands are considered characteristic of the anti-parallel beta-sheet structures. The band at 1660 cm^-1^ may be assigned to alpha-helix secondary structures. The random structures and beta-turns may be related to the bands at 1650 and in the 1670–1685 cm^-1^ range, respectively [63].

### FTIR analysis of absorbency ratios of secondary structure components

**Fig 4:**
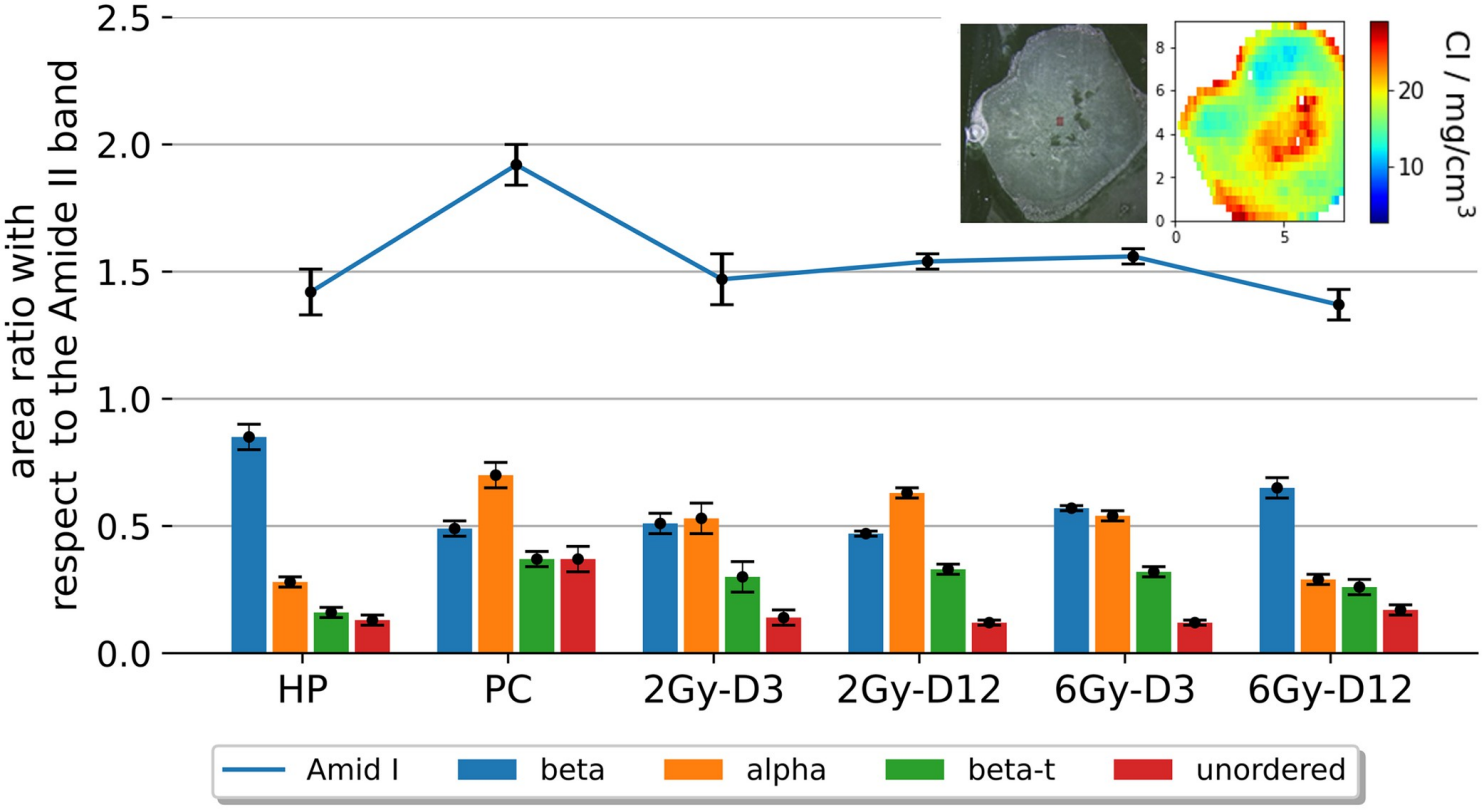
Absorbance ratios of the secondary structure contributions with respect to the amide II band for benign (HP) and malignant (PC), as well as treated tissues (2Gy- D3, 2Gy- D12, 6Gy- D3, 6Gy- D12) (n = 900 for each tissue type). The ratio of the total area of the amide I band to the amide II band is shown by the blue line. The position and area of the FTIR measurement with respect to the sample cross section is shown in the inset for the case of 6Gy-D12. The left part shows the microscopic image of the tissue section used for FTIR measurements, followed by the Cl distribution as determined from XRF on an adjacent tissue section.

The FTIR measurements have been performed on adjacent slices originating from the same tumour, as for the XRF measurements. This allows a guided FTIR measurement with significantly smaller measurement area compared to the heterogeneity observed from the XRF measurements. As an example, the position and area of the FTIR measurement relative to the sample cross section is shown in the inset of Fig 4 for the case of the 6Gy-D12 sample. The inset shows a microscopic image of the cross section accompanied by the elemental distribution of Cl determined by XRF on a parallel slice.

In order to interpret the spectral differences in terms of biomolecular changes in the tissues, a previously developed radiometric approach [23, 64–66], has been adopted for analysing FTIR spectra for the different treatment conditions listen in Table 1. To this end ratios between the secondary structure peak areas were calculated, grouped according to the different structures and to the area of the entire amide II peak at 1540 cm^-1^. Gauss-Lorentzian-shaped components were used for deconvolving the amide I band. The results are shown in Fig 4 and show that the total area of the amide I band with respect to the amide II band (blue line) was increased for the untreated PDAC tissue (PC) (1.92±0.08) compared to the healthy pancreas (HP) (1.42±0.09) and all treated PDAC tissues (1.37±0.06 to 1.56±0.03).

The amide I contribution in the PC case came mainly from random structures. Beta-s structures was 0.49±0.03 in the PC tissue, as well as in the 2Gy-D3, 2Gy-D12, 6Gy-D3 tissues. These structures were increased in the HP and 6Gy-D12 exposed PDAC tissue (with 0.85±0.05 and with 0.65±0.04 respectively). A reciprocal regulation is seen in the alpha structures. These are seen to be increased levels in sham exposed (0.7±0.05) and 2Gy-Day3 (0.53±0.06), 2Gy-D12 (0.63±0.02) and 6Gy-D3 (0.54±0.02) pancreatic cancer tissue, but they reduce in HP tissue (0.28±0.02) and 6G-Day12 KPC tumours (0.29±0.04). Unordered proteins are seen at high levels only in PC tissue (0.37±0.03), whereas beta-t structures are seen elevated in all tissues (approximately 0.32—0.37) except the normal pancreatic tissue (0.16±0.02) and the pancreatic cancer tissue exposed to 6 Gy and assessed 12 days after treatment (0.26±0.03). These results show that normal pancreas and pancreatic tumour can be differentiated based on their amide I (beta-s, alpha and beta-t) content and amide I/II ratio with pancreatic tumours having higher levels of alpha, beta-t and amide I and lower levels of beta-s than normal pancreas.

### RF-XRF of dried tissue sections of healthy pancreas and KPC tumours

**Fig 5:**
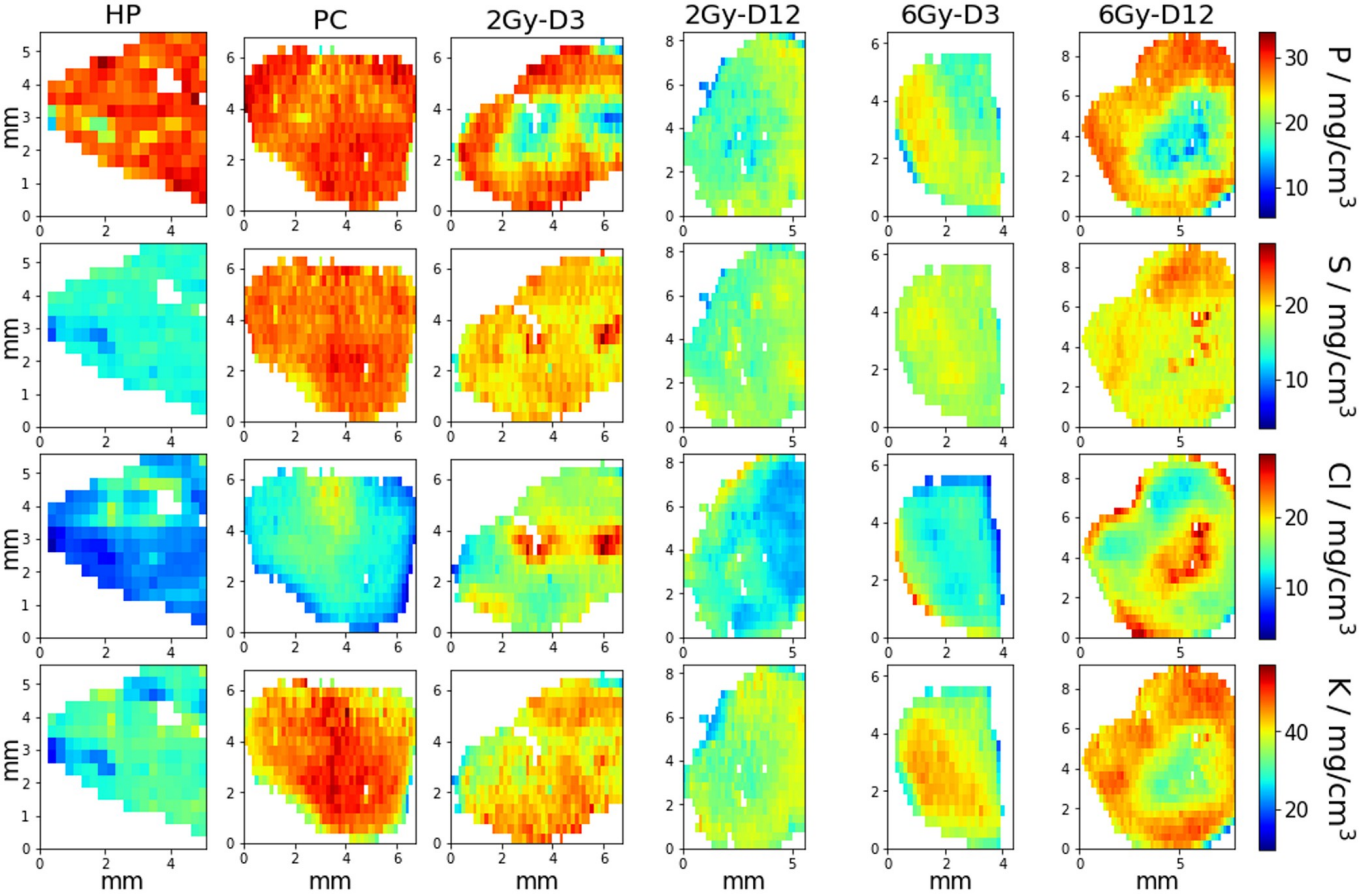
RF-XRF elemental concentration maps of phosphorus (P), sulfur (S), chloride (Cl) and potassium (K).

**Fig 6:**
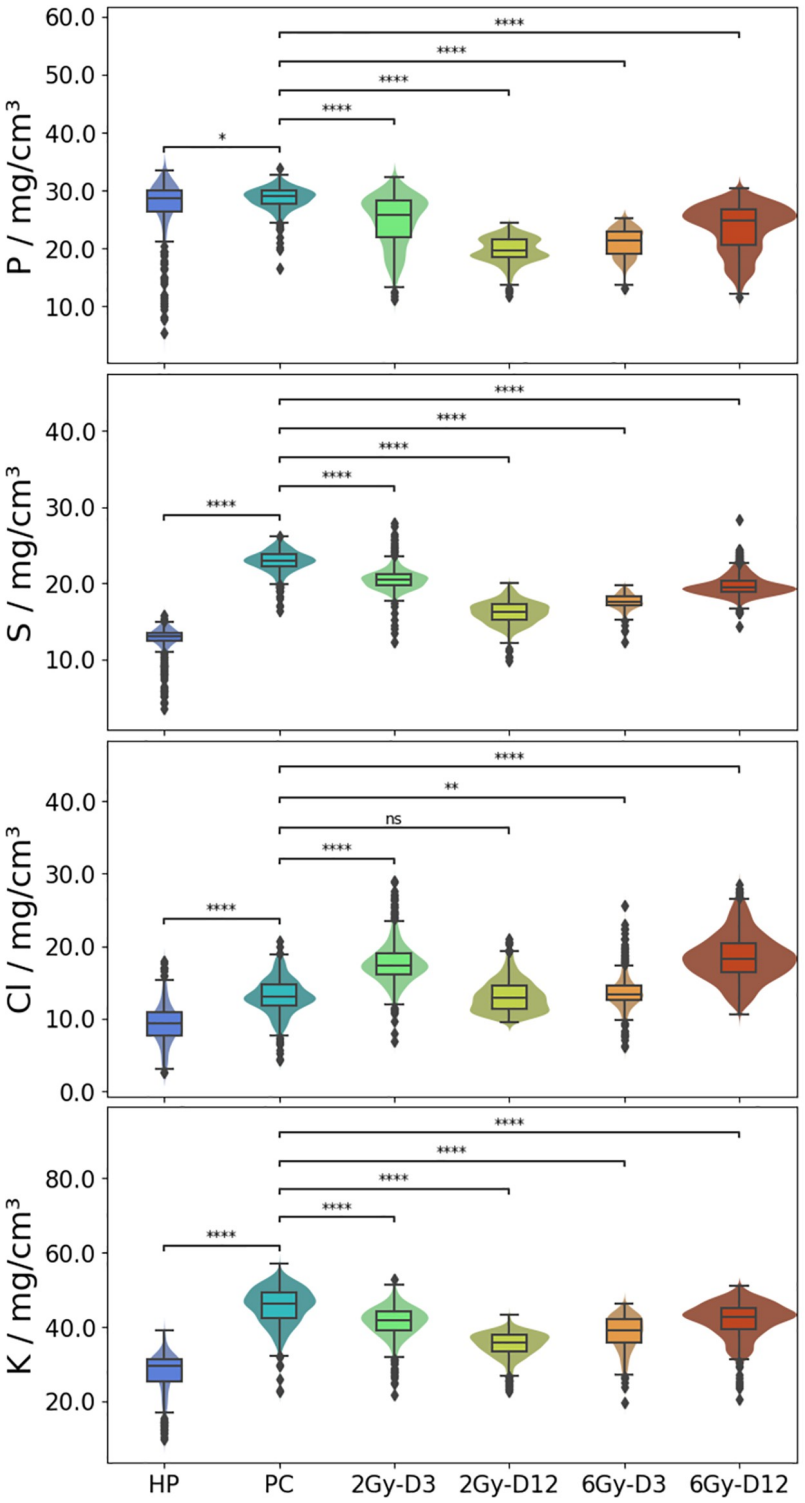
Box plots and violin plots of elemental concentration of phosphorus (P), sulfur (S), chloride (Cl) and potassium (K) showing the occurrence distribution of the concentrations throughout the measured tissue sample area (see Fig 5).

During the RF-XRF measurements in the tender X-ray range, several mm^2^-sized maps for phosphorus (P), sulfur (S), chloride (Cl), and potassium (K) of 2Gy-D3, 2Gy-D12, 6Gy-D3, 6Gy-D12 tumours, PC and HP tissues were obtained (Fig 5). These maps show the spatial distribution of concentrations of the elements within the tissue sections. The median concentrations of P, S, Cl and K in these tissues are shown in Table 4. Violin plots of these results show the statistical significances when the elemental concentrations are compared between different samples (Fig 6). For example, higher concentrations of S and K were seen in pancreatic tumours (22.9±6.2 mg/cm^3^ and 46.3±12.4 mg/cm^3^, respectively) then in healthy pancreas (13.0±3.3 mg/cm^3^ and 29.6±7.6 mg/cm^3^, respectively). Treatment of pancreatic tumours with 2 Gy and 6 Gy caused a statistically significant decrease in the concentrations of S and K compared to sham-exposed pancreatic tumours with the highest decreases seen in 2 Gy irradiated tumours 12 days after exposure (16.3±4.2 mg/cm^3^ and 35.8±9.2 mg/cm^3^ respectively), and 6 Gy treated KPCs 3 days after exposure (17.52±4.8 mg/cm^3^ and 39.2±10.5 mg/cm^3^ respectively). Although the levels of S and K in these irradiated tumours did not reach the levels seen in healthy pancreas at any of the time points investigated, a decrease was seen compared to sham-exposed tumours. The data suggest that non-destructive quantitative elemental analysis is highly sensitive to changes in the elemental compositions of tissues and that S and K could be used to differentiate healthy pancreatic tissue from PDAC tumours.

**Table 4:** Elemental concentrations (*C* in mg/cm^3^) and tissue section thicknesses (*d* in *μ*m) determined by RF-XRF. Displayed are the medians (med) ±combined standard uncertainty (csu) and the means ±standard deviation of the concentration distributions of P, S, C and K of all analysed tissue samples shown in Fig 5.

Sample		HP	PC	6Gy-D12	6Gy-D3	2Gy-D12	2Gy-D3
**N**		271	599	951	306	659	577
**C(P)**	med±csu	28.6±7.3	28.9±7.7	24.8±6.4	21.4±5.7	19.6±5	25.8±6.8
	mean±std	26.8±5.4	28.7±1.9	23.6±4.0	21.0±2.5	19.7±2.2	24.8±4.4
**C(S)**	med±csu	13.0±3.3	22.9±6.2	19.5±5.1	17.52±4.8	16.3±4.2	20.5±5.5
	mean±std	12.3±2.2	22.9±1.4	19.7±1.4	17.6±1.0	16.2±1.5	20.5±1.5
**C(Cl)**	med±csu	9.3±2.4	12.9±3.5	18.3±4.7	13.4±3.6	12.9±3.3	17.4±4.7
	mean±std	9.3±2.9	13.1±2.4	18.5±3.2	13.7±2.7	13.1±2.2	17.7±2.9
**C(K)**	med±csu	29.6±7.6	46.3±12.4	42.8±11	39.2±10.5	35.8±9.2	41.9±11.2
	mean±std	27.6±5.6	45.7±5.0	42.0±4.6	38.4±4.6	35.2±3.5	41.3±4.3
**d**	med±csu	1.99±0.52	1.15±0.31	1.6±0.4	1.1±0.3	1.9±0.5	1.2±0.3

The spatial distribution of P, S, Cl and K was homogeneous in HP and PC tissue but not in some of the treated tumours, for example those treated with 3 Gy (3 days after exposure) and 6 Gy (12 days after exposure) (Fig 5). In these tumours, areas of high Cl concentrations were co-localized with areas of low concentrations of P and K and vice-versa. All treated tissue sections demonstrated a reciprocal co-localization of P and Cl concentrations. The biological significance of these observations is unclear. Lowered concentrations of P compared to sham-exposed pancreatic tumours and healthy pancreas (both with ∼ 28±8 mg/cm^3^ of P) were seen in tumours exposed to 2 and 6 Gy (range from 19.6±5 to 25.8±6.8 mg/cm^3^).

#### Sample aging and stability (NEXAFS)

Although RF-XRF is a supposedly non-destructive method, changes in the chemical species of the sample can occur since the applied excitation radiation can cause changes in binding states of atoms under investigation by breaking molecular bonds. In biological samples, changes can also appear due to storage or aging effects. To monitor these effects, Near-Edge X-ray Absorption Fine Structure (NEXAFS) investigations were conducted. NEXAFS spectroscopy allows probing the chemical states of the elements of interest, and therefore to address the possibility of excitation radiation-induced changes of chemical binding states.

**Fig 7:**
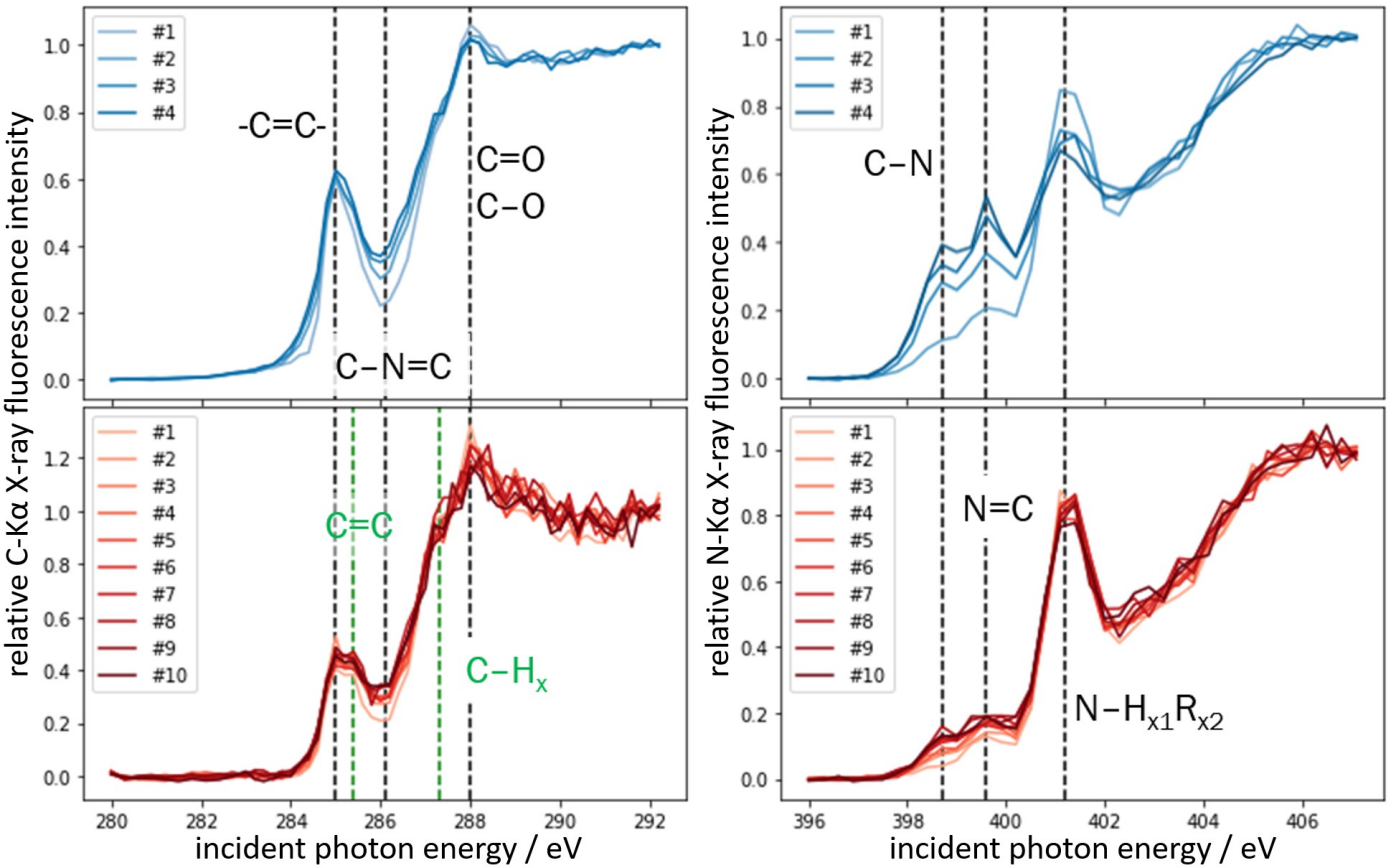
Chemical sample stability investigation results. Normalised C K-edge (left column) and N K-edge (right column) NEXAFS spectra of two adjacent tissue sections of the 6Gy-D12 tumour measured one month (top row, blue) and six months (bottom row, red) post preparation. Differences between NEXAFS features indicate changes in the overall binding states of all carbon or nitrogen atoms in the sample volume analysed. The comparison between spectra in the same figure shows the effect of X-ray radiation induced changes. The comparison between top line and bottom-line figures shows the effect of sample aging. Prior to the NEXAFS measurements no RF-XRF investigations of the samples were conducted. Each of the tissues was measured 12 hours after being transferred into the UHV measurement chamber. The measurement repetition number at the same position is indicated by ‘#’. The dotted lines in the C-K edge spectra are positioned at 285 eV (C = C), 285.4 eV (C = C), 286.1 eV (C≡N), 287.3 eV (C-H), and 288 eV (C = O) (left to right). In the N-K edge spectra, the dotted lines are positioned at 398.7 eV (N = C), 399.6 eV (N≡C), and 401.2 eV (N-H) [67–70].

X-ray irradiation induced changes of the chemical binding states of carbon and nitrogen have been assessed on 2 adjacent tissue sections of the 6Gy-D12 tumour. Repeated NEXAFS measurements in the same sample position of one post sample preparation showed the impact of X-ray radiation on the chemical composition of the sample (see Fig 7, top row). The excitation radiation caused the additional formation of aldehyde groups (R–CH = O), which have π* resonances at 286.1 eV [67]. In the spectra of the N K-edge, a distortion of N–H bonds (π* resonances at 401.2 eV), and the formation of C = N (398.7 eV) and C≡N (399.6 eV) bonds can be observed. Repeated NEXAFS measurements on a adjacent tissue section five months later (see Fig 7, bottom row) display a similar effect of the excitation radiation, but less pronounced. Overall, the NEXAFS measurements show that the X-ray excitation radiation changes the chemical composition of fresh samples, but this appears to be less in aged samples. The most important changes of the chemical composition in the sample are, however, induced by aging processes, these are not localized, but affect the chemical composition of the whole sample.

## Discussion

State-of-the-art cancer detection, characterisation, and response to treatment monitoring are strongly based on molecular biomarker analysis. Despite advances in digital imaging, the lack of reliable and practical quantitative histological techniques hampers the ability to quantify biomarker changes in tissues. The applicability of multimodal / hybrid metrological approaches depends on the ability to relate the quantities that underpin the respective measurands [71]. Ideally, independent quantities are derived for which a physical or chemical relationship exists, and correlations can be identified. If this relationship is unknown, correlations between different measurands (or quantities) may be revealed or at least may indicate behaviours related to certain sample properties seen. For an all-encompassing characterization of complex biological samples, the combination of molecular and elemental biomarkers is a promising approach, as two independent quantities can be used to describe the tissue state. Ultimately, this type of information can be linked to the underlying biology of tissues, and thus, a causal relationship between them can be established. Moreover, a quantity linked to the (mostly qualitative) results of a molecular characterization technique can provide reference values. These values could allow absolute comparison between results, potentially significantly improving cancer detection. This would be particularly useful in the case of pancreatic cancer where the majority of patients (> 80%) present with the disease at an advanced stage, and where novel methods that will enable the earlier detection of the disease are of urgent clinical need. In this study we assessed the ability of combined investigations of FTIR and RF-XRF to identify elemental indicators for different tissue states, to discriminate between healthy tissue and cancer, and to assess their sensitivity for monitoring radiotherapy treatment effects.

The success of a multi-method investigation depends on the complementary nature of the chosen methods. The chosen methods should preferably have independent measurands including their derived quantities to prevent influencing one another’s results. This criterium is met for the combination of FTIR and XRF [72, 73], reflecting different chemical quantities, i.e. elemental concentration and molecular arrangement (binding state). The quality of the alignment of independently acquired results depends on the lateral resolution and information depth of each of the methods to ensure the overlap or even full equivalence of the probed sample volume. With a dried tissue sample thickness of 1 *μm* to 2 *μm* the depth compatibility of both methods was ensured. Based on the requirements of sample preparation for each method, the preparation protocol must be tuned, and the right substrates chosen. An overview of these considerations regarding FTIR and RF-XRF is shown in Table 5. In view of the complexity and limited stability of biological sample systems there is evident need for flexible and highly reliable chemical analysis. On the one hand, flexibility is ensured by the tunability of experimental or instrumental parameters such as the lateral probing resolution. On the other hand, most analytical techniques require calibration specimens, the spatial elemental matrix distribution of which is similar to that of the biological sample of interest, in order to ensure low levels of uncertainty. This challenge can be addressed in two ways: the provision of appropriate calibration samples, or the provision of an analytical method traceable to the SI.

**Table 5:** Comparison of capabilities and constraints of RF-XRF and FTIR in terms of their sample preparation requirements for multi-method investigations of similar and identical biological tissue samples.

	FTIR	RF-XRF
**Analytical information**	laterally resolved vibrational state of the molecular ensemble	laterally resolved elemental concentrations
**Type of information**	qualitative + quantitative	quantitative (traceable to SI)
**Measurand**	absorption	emitted fluorescence intensity
**Quantity**	amount of substance (mol m^-3^)	elemental mass per unit area (g cm^-2^)
**Possible additional information**	molecular composition	chemical speciation (NEXAFS), sample thickness (X-ray transmission)
**Lateral resolution**	ca. 10 *μ*m (30 nm for super-resolution methods)	90 nm to 0.5 mm
**Information depth**	several micrometres (100 nm for super-resolution methods)	0.4 *μ*m (carbon) to 0.22 mm (potassium) for light matrices
**Substrate requirements**	substrates with reflective coating	should not contain elements of interest, homogeneous elemental composition, ideally quartz or Si-wafer
**Experimental atmosphere**	ambient	vacuum
**Destructiveness**	non-destructive	non-destructive towards the elemental sample composition, can influence the chemical species
**Sample preparation adjustment**	snap freezing of tumours / organs after extraction by immersion in liquid nitrogen, cutting of 10 μm sections using a cryotome, placement of sections on quartz or coated glass slides, air drying of the samples provides enough adhesion to the substrate, no application of fixation or embedding materials/chemicals

The latter is the case for reference-free XRF and reflects its value and high impact for the characterisation of biological samples. The possibility of working without the need for calibration samples ensures the high reliability of the chemical analysis provided by this method. It allows the reliable quantification of elemental concentrations with well-known uncertainty. Using synchrotron radiation, its detection limits are in the parts per billion range. The lateral resolution of XRF can be tuned dynamically to meet application requirements from the sub-mm to the sub-μm regime. In addition, to specialised synchrotron facilities which can measure under different atmospheric conditions and temperatures, a wide range of efficient laboratory measurement setups for elemental mapping are available, allowing higher sample throughput, and more flexible long-time access. Regarding the sample preparation, no extensive staining or labeling protocols are needed. More important is the choice of the flat and homogeneous substrate, which ideally contains no elements of interest. When measuring light elements such as C and N, the absorption of the fluorescence by the biological sample must be taken into account. Free standing samples, which would allow experimental transmission measurements to determine the absorption coefficient would be preferable. Alternatively, the samples could be mounted on thin substrates that would also allow such measurements. As FTIR relies on standard sized and coated substrates, this could not be achieved in this study, resulting in higher uncertainties for the reference-free quantification. XRF does not change the elemental composition of the sample, but it may affect binding states, particularly when examining the primary matrix elements of biological tissues such as carbon and nitrogen (see Fig 7). The likelihood of modifying chemical binding states through X-ray radiation depends on the difference between the energy of the incident photon and the absorption-edge energy of the element of interest as well as from the absolute radiation dose (or radiant power) applied to the sample. Therefore, it is recommended to perform molecular composition probing with FTIR before conducting NEXAFS experiments on main matrix elements. However, this restriction applies less to RF-XRF investigations of higher Z elements.

This case study shows the potential and challenges of the quantification of trace elements in dried biological tissue samples using RF-XRF. The results of the RF-XRF investigations demonstrated that healthy pancreatic and malignant pancreatic carcinoma tissues can be discriminated using their elemental composition in S and K with significantly higher levels for both elements seen in malignant tissue. Furthermore, this method was applied for monitoring the regulation of these elements after low dosage radiotherapy treatment of PDAC with results showing a decrease in both these elements after radiotherapy (see Fig 5). The reduction in S that was seen in irradiated tumours is suggestive of a reduction in protein synthesis since S comprises part of the aminoacid cysteine [74], and is also found in proteins containing disulphide bonds [75]. K on the other hand, is the most abundant cation found in intracellular fluid and so its reduction in irradiated tumours compared to sham exposed tumours is suggestive of a reduced number of intact tumour cells in the treated tissues. Regulation of P in irradiated KPC tumours was also seen, with a significant P reduction detected in tumour tissues exposed to 2 Gy (12 days after treatment) and 6 Gy (both 3 and 12 days after treatment). This may be suggestive of the irradiation-induced loss of cells with concomitant loss of DNA, cell cycle inhibition and reduced cellular metabolism and catalytic activity in treated tissues since P is a fundamental element contained in cellular DNA and ATP [76]. These results provide insights into the anti-cancer effect of radiation treatments and the regulation of biomarkers at the elemental level. These differences would otherwise be difficult to detect.

The analysis of KPC and healthy pancreas tissue sections of murine subjects by FTIR followed with RF-XRF revealed coherent results. As treated cancer cells do not directly transform back to healthy ones, the additional elemental analysis can provide additional insight towards the actual state of the cancer tissue and its reaction to radiotherapy. Through the combined analysis of FTIR and RF-XRF it has been demonstrated that both methods provide independent indicators for malignant tissue regions, leading to enhanced confidence in our findings. Both methods were capable of discriminating healthy tissues from KPC tumours. In addition, the analysis of irradiated KPC tumours revealed a systematic change of the molecular sample composition towards the composition of the healthy tissue with higher radiation doses and longer reaction times (see Fig 4). Amongst the biomarkers detected were the amide bonds. Amide bonds are known for maintaining stability and proclivity in forming resonating structures, which in turn influence secondary structure adoption and biological activity [77]. Further band positions, band widths (full-width-at-half-maximum, FWHM) and band shifts of amide bonds may represent an important indicator for molecular structural (re-) organisations, occurring either in the course of interaction(s) or under physico-chemical treatments such as X-ray irradiation. FTIR characterization of amides I and II indicated that the secondary structures of proteins were significantly different between the healthy and malignant pancreas and further modified by the treatment of PDACs towards a phenotype seen in healthy pancreas. For example sham-exposed pancreatic tumour sections and those exposed to either low levels of radiation (2 Gy) or assessed at early time points (3 days post treatment) retained their tumour phenotype showing decreased beta-s and increased alpha and beta-t compared to the healthy pancreas. In contrast, when a pancreatic tumour was exposed to a radiation dose of 6 Gy and was assessed at a late time point (12 days post treatment), which allowed the anti-cancer effect of this radiation dose to fully manifest, the regulation of beta-s, alpha and beta-t, was seen to revert to levels seen in the healthy pancreas (see Fig 4). These results suggest that beta-s, beta-t and alpha could become treatment biomarker candidates to denote the effects of treatment. Also, the significance of the increased levels of amide I seen in the untreated pancreatic tumours compared to all other tissues is a result that warrants further investigation. Future studies using treatments that result in complete tumour growth control would show whether these elements could become treatment biomarkers to denote the loss of the carcinogenic potential of the tissue.

There are a number of limitations to our study. A low number of samples was used since the aim was to provide proof of concept. To fully assess the effect of radiotherapy additional investigations should be performed including a longitudinal assessment of tumour growth. Another limitation comes from the limited area of the sample covered by FTIR. FTIR mapping that covers the same sample area as with RF-XRF is prohibitively time-consuming. To overcome this, a data fusion approach between FTIR and RF-XRF was carried out. Relevant regions were selected based on RF-XRF, so that FTIR can be most efficiently applied and so that biological variability over the measurement region does not mask specific spectral changes. Therefore, the combination with RF-XRF is especially advantageous in order to pre-select areas potentially containing molecular biomarkers, or to exclude the abundant uninformative and uncorrelated data to selectively identify biomarkers. The importance is highlighted by the heterogeneity observed by RF-XRF for which an unguided small area FTIR characterisation may lead to a less conclusive results. As X-ray irradiation can alternate the chemical speciation of elements in the sample, such a pre-selection of areas is not recommended for the analysis on the same sample but rather on adjacent slices of the same tumour.

**Fig 8:**
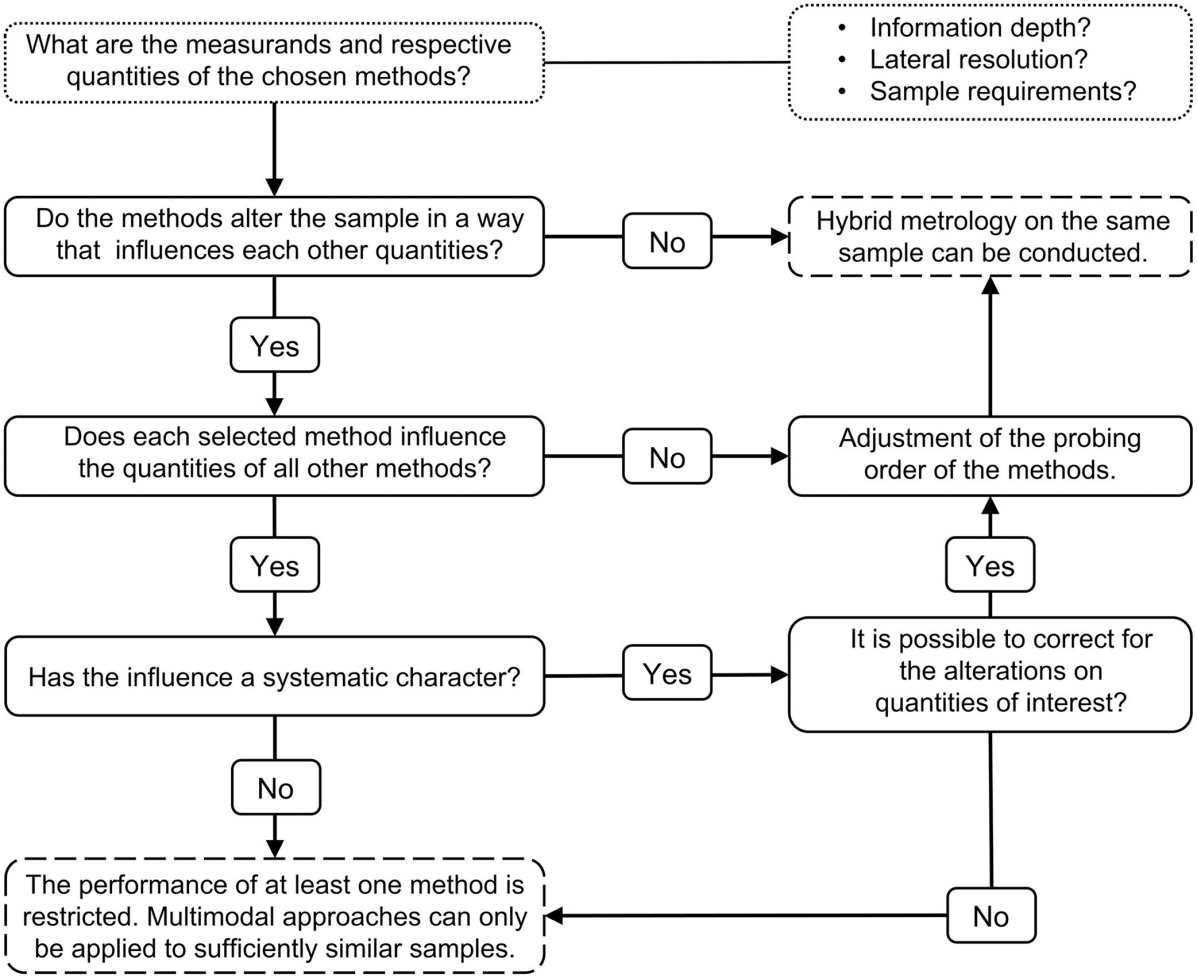
Flow chart for the design of multi-method or hybrid studies.

Fig 8 shows our recommendations for the design of multi-method/hybrid studies. The effect of the methods used regarding the influence of the measurands and respective quantities should always be examined prior to the correlation of results. Accordingly, the order of probing by different methods must be adjusted. If the methods do not influence each other’s measurands and preferable quantities, hybrid investigations of the same sample can be successfully undertaken, as demonstrated by combining FTIR and RF-XRF. Otherwise, the influence of the methods on the obtained results must be thoroughly assessed. If the influence is systematic, it can be considered, otherwise correlative studies on sufficiently similar samples must be undertaken.

## Conclusion

Multimodal/hybrid metrology investigations of biomedical samples including SI traceable XRF provide the opportunity to acquire molecular and elemental information about the same sample. Due to both the high variability of biological samples and the deficiency of reference materials or appropriate calibration samples, the non-destructive elemental quantification by means of RF-XRF can be expected to substantially contribute to a reliable cancer detection and treatment monitoring in research and patient care. RF-XRF does not require elaborate sample preparation techniques and can be applied to samples previously analysed by already established techniques such as FTIR and therefore qualifies for multimodal or hybrid metrology investigations of biomedical samples. The added value of the combined investigations of FTIR and XRF is the acquisition of independent molecular and quantitative elemental information on the same sample, allowing for a more reliable assessment of the metabolic state of the tissue. Using this approach we show for the first time that a) biological molecules including amide, S, and K can be used to differentiate PDAC tumours from healthy pancreas, b) the response of PDAC to radiotherapy is associated with a reduction of S and K, c) The response of PDAC to radiotherapy is associated with a reduction of amide I to levels seen in healthy pancreas. Therefore, the combination of molecular mapping with FTIR and quantitative elemental mapping with RF-XRF supports the identification of elemental and molecular markers and allow for further insights into the response of tumours to treatments, thus allowing for a more target-oriented molecular screening of the samples. The quantification of elements using the techniques described in this study might be relevant to the earlier detection of pancreatic carcinomas, for example where pre-cancerous cystic lesions are formed, or these could be used as prognostic biomarkers for the effectiveness of treatments. In both cases clinicians will have a powerful new tool to use for the earlier detection of disease or to choose the best possible course of treatment. For a disease such as pancreatic cancer both these outcomes can be a matter of life or death for some patients.

## Supporting information

S1 Table Record of treatments. (PDF)

S1 File The ARRIVE guidelines 2.0 checklist. (PDF)
