# Supplementary material for: Complementary techniques for the reliable characterisation of tissue samples: A case study on pancreatic tumours analysed by means of X-ray fluorescence analysis and IR spectroscopy
Source: PLoS One. 2024 Sep 4;19(9):e0306795. doi: 10.1371/journal.pone.0306795 (PMC11373814; doi:10.1371/journal.pone.0306795)
Supplement: S1 Table — (PDF) [file pone.0306795.s001.pdf]

Table S1: Record of treatments

Animal ID	Site	No. beams	Beam angles	Collimator	Dose (Gy):	Date:
98098_4_2Gy	SC	2	-10, 170	10x10	2	2021-04-13
98098_5_2Gy	SC	2	-10, 170	10x10	2	2021-04-13
98098_2	SC	-	-	-	control	-
98099_2_6Gy	SC	2	-10, 170	10x10	6	2021-04-13
98099_4_6Gy	SC	2	-10,170	10x10	6	2021-04-13
98099_5	SC	-	-	-	control	-
